# Potential correlation between chronic pain and amyloid beta in Alzheimer's disease

**DOI:** 10.3389/fpain.2026.1799860

**Published:** 2026-03-23

**Authors:** Yanying Liu, Xiao-Ping Wang

**Affiliations:** 1 Department of Basic Medicine, School of Medicine, Qingdao Huanghai University, Qingdao, China; 2 Department of Neurology, Shanghai Jiao Tong University Medical School, Shanghai, China

**Keywords:** Alzheimer's disease, amyloid beta, chronic pain, cognitive decline, pain

## Abstract

Pain refers to an unpleasant sensory and emotional experience associated with actual or potential tissue damage. Chronic pain is a common symptom among Alzheimer's disease (AD) patients. Due to the cognitive impairment characteristic of mid-to-late-stage AD, many AD patients fail to receive timely pain relief, leading to worsening of the disease. Understanding the relationship between chronic pain and the pathological progression of AD is crucial. Previous studies have confirmed a close correlation between pain occurrence and the metabolism of amyloid beta (A*β*) protein, one of the hallmark pathological features of AD. This article provides an overview of recent research progress on the interaction between pain and A*β*, analyzes its molecular mechanisms, and offers new research insights for effectively alleviating pain in AD patients and preventing or treating AD.

## 1 Introduction

Pain, a complex psychobiological process triggered by internal and external harmful stimuli, is not only a symptom of disease but also a condition in itself. Over the years, significant progress has been made in the medical field regarding the study of pain and its mechanisms (1, 2). However, due to the intricate factors involved in the formation and maintenance of pain, many unknown areas remain, necessitating further in-depth research. There are numerous methods for classifying pain. According to the duration of pain, it can be divided into acute pain and chronic pain. Acute pain is usually a sudden onset of pain with a short duration (less than 1 month) caused by illness or trauma, and is self-limiting. When the cause of pain is removed, the pain can be relieved. If it does not relieve, it can develop into chronic pain. Chronic pain is commonly a symptom of some chronic diseases, which lasts for more than 1-3 months and often recurs.

Alzheimer's disease (AD) is a common age-related neurodegenerative disorder that affects behavior, learning, memory, and other cognitive functions in elderly individuals (3). With the aging of the global population, the incidence rate of AD is on the rise year by year, which has brought a heavy burden to families and society. Although AD has been discovered and studied for over a century, its unclear etiology has hindered effective prevention and treatment. In the early stages of AD, patients can clearly describe the chronic pain they often experience as their neurological function is not yet impaired. However, in the later stages of AD, cognitive decline caused by impaired neurological function leads to inaccurate pain feedback, which in turn accelerates the patient's pathological progression (4). Amyloid beta (A*β*) is the main component of senile plaques, a characteristic pathological structure of AD, and there is a close correlation between abnormal aggregation of this protein and pain. Therefore, clarifying the relationship between chronic pain and A*β* will provide valuable references for the prevention and treatment of AD. Unfortunately, the relationship between chronic pain and AD incidence remains unclear to this day (4).

This article summarizes recent research advancements in the study of pain and AD, focusing on the relationship between pain and the pathological structural protein A*β*, as well as its molecular mechanisms. It provides new research insights for the prevention and treatment of chronic pain and AD.

## 2 Pain and its modulation mechanism

**Figure 1:**
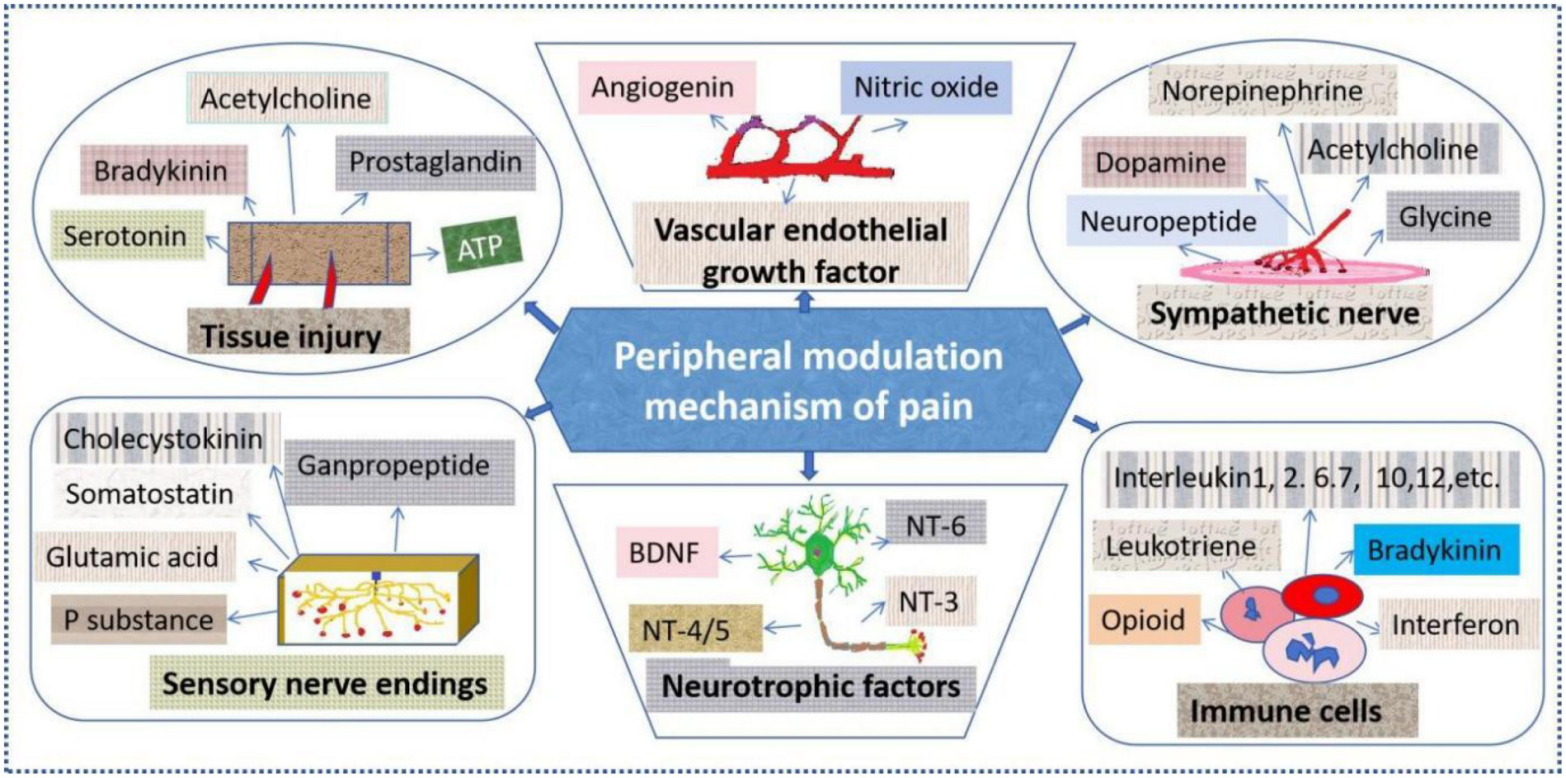
Possible mechanisms of peripheral modulation in pain. Harmful stimuli induce the production and release of various chemicals or cytokines in peripheral tissues, which participate in the activation or regulation of nociceptors. It contains prostaglandins, serotonin, bradykinin, and other substances produced locally in stimulated tissues, as well as bioactive substances such as P substances and somatostatin at the sensory nerve endings. Moreover, vascular endothelial growth factor, nitric oxide, and various neurotrophic factors are involved in the blood vessels and brain tissues. In addition, other substances, including glycine, dopamine, neuropeptides, various cytokines, leukotrienes, interferons, etc., are produced in immune cells and the sympathetic nervous system.

Pain refers to an unpleasant feeling or experience in the body that is accompanied by actual or potential tissue damage. It is usually a protective defense response of the body against harmful stimuli (5). Under physiological conditions, when body tissues are damaged, pain signals are converted from the original injury sites into nerve impulses and transmitted to the central nervous system. After information processing and integration, they ultimately cause pain reactions. Pain modulation can be divided into central and peripheral mechanisms. The peripheral mechanism refers to the generation and release of various chemicals or cytokines in peripheral tissues in response to nociceptive stimuli, which participate in the activation or modulation of nociceptors (6, 7). As shown in Figure 1, these media directly activate receptors to induce pain sensation. The understanding of the central mechanism of pain modulation has gone through various theories. (1) Gate control theory. This theory suggests that the gate of influence includes three aspects: input fibers, intramedullary staged response, and descending control. Promoted research and development in physiology, pharmacology, and therapy (8). (2) Endogenous pain modulation system. The involvement of opioid peptides (endorphins, enkephalins, and dynorphins), serotonin, acetylcholine, and vasopressin is the basis of endogenous pain modulation (9). (3) Plasticity changes/central sensitization (10). Its main manifestations include sensitization of spinal dorsal horn neurons, functional inhibition of the spinal cord inhibitory regulatory system, sensitization of upper spinal cord neurons, and changes in the activity of the descending regulatory system or activation of glial cells, which play a key role in the generation and maintenance of pain. In summary, the generation and modulation of pain are complex processes that are regulated by numerous factors. Currently, following the development and application of advanced scientific technologies such as positron emission tomography, single electron emission tomography, and functional magnetic resonance imaging in modern medicine and life sciences, the effects of pain can be directly and clearly observed in different brain regions. In particular, the development and application of advanced technologies such as bioinformatics (11) and network pharmacology (12) might provide technical support for unraveling the mysteries of pain and pain-related disease mechanisms, and effectively preventing and treating certain diseases.

## 3 Alzheimer's disease

Alzheimer's disease is a central nervous system degenerative disorder characterized by progressive cognitive impairment and behavioral damage with insidious onset. According to the age of onset, AD can be divided into early-onset familial AD and late-onset AD. In clinical practice, more than 90% of patients develop the disease in old age or pre-old age. Although the etiology of AD is unclear, numerous studies have confirmed that genetic and environmental factors are closely related to the onset of AD (13). The characteristic pathological changes of AD include senile plaques formed by A*β* aggregation, neurofibrillary tangles formed by highly phosphorylated tau protein, and inflammation. Therefore, A*β* and tau have become key drug targets for AD treatment (14), and anti-inflammation has become one of the approaches for AD therapy (15). Recent studies have shown that the plasma level of the A*β*42/40 ratio is sensitive to early brain amyloid accumulation and predicts the risk of cognitive decline in the AD spectrum, as well as gut microbiota metabolites (16) could be potential therapeutic targets for AD (17). Similar reports suggest that the possibility of A*β* deposition begins in olfactory neurons and then extends from the nasal cavity to the central nervous system, which is closely related to the onset of AD (18). These studies support the importance of A*β* in the pathogenesis of AD. Recently, Sun et al. (19) have adopted multimodal data to accurately predict cerebrospinal fluid A*β* protein for early detection of AD. Hossain et al. (20) analyzed the potential of Mitragyna speciosa in treating AD through network pharmacology, molecular docking, and *in vitro* insights, providing a valuable tool for early detection of AD.

## 4 The relationship between pain and AD

Pain is one of the main symptoms of many human diseases, such as hip joint disease (21, 22), nerve damage (23), migraine and degenerative cervical spinal cord disease (24, 25). Chronic pain is commonly present in patients with AD and is positively correlated with the severity of cognitive impairment, affecting their quality of life. At present, there is insufficient research evidence to suggest that pain can lead to the occurrence of AD. However, in the early and middle stages of AD, more than 50% of patients often feel chronic pain. The fact that appropriate anti-pain medication treatment (26) or pain relief measures (27) can improve the symptoms and prognosis of AD patients indicates a complex relationship between pain and AD. The pain can disrupt various cognitive abilities, including learning and attention, problem-solving skills, language communication abilities, etc., in AD patients. Zhao et al. reported that pain patients had a higher risk of developing dementia and cognitive impairment, and accelerated brain aging increases the risk of death (28). In addition, Cowan et al. found that AD patients required higher stimulus to feel the pain before the injury was identified and reported due to reduced pain sensitivity (29). These studies collectively indicate that as AD progresses, AD patients’ ability to recognize and self-report pain decreases. The decrease in pain sensitivity, although reducing interference with brain cognitive function, is closely related to the progression of AD.

Recent studies have shown a close correlation between chronic pain and biomarkers of neurodegeneration, microglial activation, inflammation, and cognitive impairment in cerebrospinal fluid (30). Hayashi et al. (31) used animal models to investigate the effect of hippocampal injury on pain perception in AD rat models induced by hippocampal injection of A*β* and Ibrutinib acid. The research results showed that compared with sham-operated rats, AD model rats exhibited significantly less pain-related behavior in the second stage of the 4% formalin test. In addition, through blue spot sensitive MRI detection and comprehensive analysis, Bell et al. found that chronic pain is associated with early affected plasma biomarkers and brain regions in AD (32). Reducing middle-aged pain and elucidating biological mechanisms may help reduce the risk of AD in older adults.

According to reports, women with a history of migraine have a significantly increased risk of developing AD. But how to solve gender related migraine problems in AD patients is a thorny issue. Fischer et al. (24) studied the response of nitroglycerin-induced experimental migraine using an AD-like triple transgenic (3 × Tg) mouse model. The research results indicate that female AD mice exhibit accelerated pain responses and reduced cognitive abilities in spatial learning and memory tasks compared to male mice, which may be related to the accelerated production of amyloid proteins in their bodies.

## 5 Research on the relationship between pain and A*β*

Chronic pain is a risk factor for AD, but its relationship with the pathology of AD is still unclear. Chronic pain can be divided into single-site chronic pain and multi-site chronic pain according to the location of occurrence (33). Research has shown that chronic pain in multiple sites rather than a single site is associated with cognitive decline and increased A*β* deposition in individuals with an increased genetic risk of AD. These findings suggest that reducing pain may lower the risk of cognitive decline and dementia.

Although the mechanisms of different types of pain are complex, it has now been proven that chronic pain may be related to the levels of A*β* in the body. Wen et al. found a correlation between the chronic pain intensity and the levels of A*β*1-42 and A*β*1-40 in cerebrospinal fluid, as well as cognitive ability, in patients with osteoarthritis after detecting their pain intensity and cognitive function (34). The levels of A*β*1-42 in patients with fibromyalgia (FM) are significantly higher than those in the healthy control group (35). The research by Bell and colleagues suggests that chronic pain is associated with elevated plasma A*β* and hippocampal atrophy. When chronic pain coexists with systemic inflammation, it may increase the risk of neurodegeneration in AD-susceptible areas (36). Since chronic pain in AD patients is associated with A*β*, early clearance of A*β* can reduce pain and the risk of developing AD. Moreover, the enhanced lymphatic function can clear metabolic waste such as amyloid beta protein and lactate from the brain parenchyma, ultimately alleviating chronic neuropathic pain (37). In addition, anti-chronic pain drugs and antispasmodics can respectively regulate the *in vivo* effects of A*β* and inhibit the aggregation as well as degradation of formed A*β* proteins (38, 39), providing new potential targets for the development of new drugs for AD. In short, the above studies have demonstrated a close correlation between the levels of A*β* and chronic pain in AD from different perspectives. We know very little about the mechanism of action of A*β* on pain, and further exploration is needed.

## 6 Molecular mechanism study on the relationship between pain and A*β*

The neurodegenerative brain regions of AD patients partially overlap with the brain regions related to pain processing, leading to abnormal responses to pain sensation in patients. The brain, especially the hippocampus, periaqueductal gray (PAG), anterior cingulate cortex (ACC), and locus coeruleus (LC), has multiple functions in cognitive, pain, and metabolic aspects. These have become neuropathological associations with comorbidities between AD and pain. A*β* is one of the characteristic pathological markers of AD, and the deposition of A*β* in the aforementioned brain regions affects the body's response to pain. The threshold of pain changes with age, and mature adults typically exhibit higher pain thresholds than young people. To clarify its mechanism, Hwang et al. studied the role of A*β* in regulating heat pain sensitivity in the dorsal root ganglion during adult maturation. The research results indicate that the role of A*β*1-42 in thermal pain sensitivity is achieved through the regulation of the LRP1/SHP2 pathway (40). This discovery provides new insights into the regulation of pain sensitivity during the maturation process. New potential therapeutic targets have been identified for age-related chronic pain management.

Although the mechanism of reduced pain sensitivity associated with AD has not been elucidated, the close correlation between A*β* and the occurrence of pain has been demonstrated by different laboratories. Cui et al. (41) studied the relationship between pain caused by sciatic nerve injury and A*β* in SD rats. The research results indicate that spinal cord A*β*1-42 acts as an endogenous analgesic peptide by regulating cytokines and the Wnt signaling pathway. This study provides a novel explanation for the reduced pain sensitivity associated with AD, which throws new light on the potential target of A*β*1-42 as an endogenous peptide for the development of novel analgesics.

Amyloid precursor protein (APP) is crucial for the pathogenesis of AD. At present, research on the role of APP and its two mammalian homologs, amyloid precursor protein 1 and 2 (APLP1, APLP2), in spinal cord injury information processing has shown that spinal cord GABA can inhibit neuronal APLP2 expression reduction, leading to neuronal injury induced microglial activation and pain sensitization, thus supporting the study of the close correlation between A*β* and pain occurrence (42).

Chronic pain patients usually have cognitive impairment in addition to changes in A*β* levels; This is particularly true in elderly patients with neurodegenerative diseases such as AD, but the mechanisms underlying this association are still unclear. Wang et al. (43) established a neuropathic pain model in 5-month-old transgenic APPswe/PS1dE9 (APP/PS1) mice by partially ligating the left sciatic nerve to investigate the relationship between chronic pain and AD. The research results indicate that chronic pain may exacerbate cognitive impairment and depression-like symptoms in APP/PS1 mice by worsening pathology associated with A*β* and tau, as well as upregulating signaling pathways involving IL-1*β* and p65. Similar reports suggest that chronic neuropathic pain and cognitive impairment may be related to the inhibition of hippocampal neurogenesis mediated by CCL2/CCR2 signaling. Therefore, treatment strategies to alleviate neuropathic pain may slow down cognitive decline in patients with AD and other neurodegenerative diseases (44). These studies have shown that inflammatory factors play a crucial role in pain regulation in AD. Recent studies have shown that the changes in nociceptive response in AD mice carrying human APP genes are related to striatal-enriched protein tyrosine phosphatase signaling (45). The study has identified the mechanism of reduced nociceptive sensitivity in AD mouse models, which can serve as a therapeutic target for improving the quality of life of AD patients.

Danshensu acid B exhibits antidepressant and analgesic effects in a pain depression comorbidity model (46) and the protective effect of ethanol extract of white wax tree bark on aluminum chloride induced AD rat model and its correlation with anti-inflammatory and antioxidant effects have demonstrated that the development of anti-neuropathic pain drugs may become one of the most promising drugs for the prevention, management, or treatment of AD and related diseases (47). Although the molecular mechanisms of these drugs are not yet clear, these studies also provide new research ideas for clarifying the relationship between chronic pain and AD.

**Figure 2:**
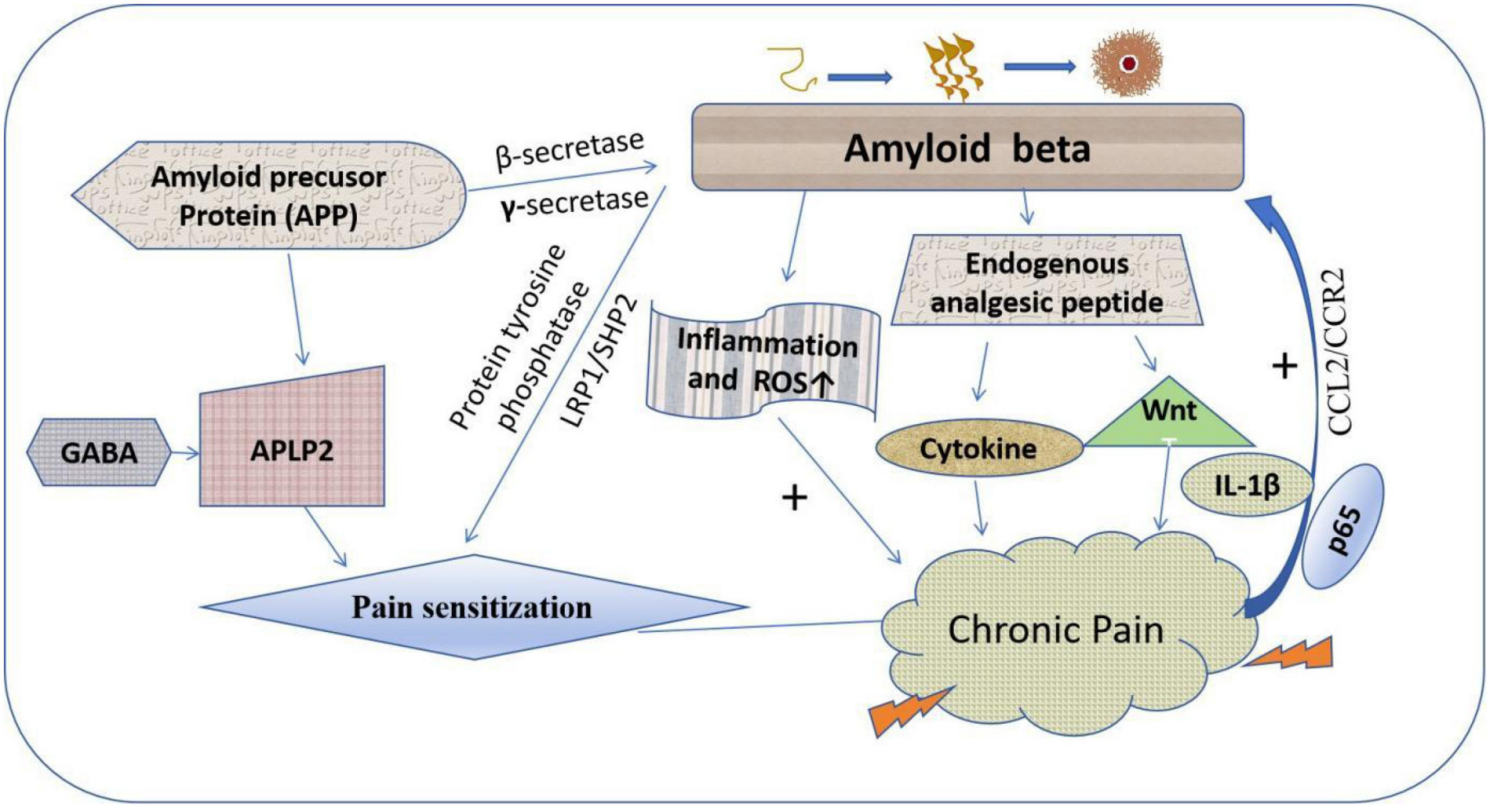
The complex regulatory relationship between A*β* and pain. A*β* can serve as an endogenous analgesic. On the one hand, it interferes with pain response by affecting the perception of pain. On the other hand, its abnormal aggregation can cause inflammation and oxidative stress, thereby increasing pain response.

In summary, there is a complex correlation between chronic pain and A*β* in AD. As shown in Figure 2, A*β* and its precursor protein APP can regulate chronic pain associated with AD through direct and/or indirect interactions. However, chronic pain in turn affects the deposition of A*β* and other pathological processes of AD.

## 7 Conclusion

Pain is a key sensation of the body in response to internal and external stimuli. Although chronic musculoskeletal pain is common in AD patients, it is left untreated due to the cognitive impairment associated with the disease. Although chronic pain in AD is related to the levels and metabolism of A*β*, timely and effective analgesic treatment may alter the levels of A*β* and cognitive abilities in the body. However, it has not had the clinical validation to date. Therefore, clarifying the relationship between A*β* and chronic pain has special meaning for whether pain management is involved in AD prevention and treatment strategies, and more research is needed in the future to verify it.
